# Best clinical practise guidance for the use of antibiotics in children: an EAPD policy document

**DOI:** 10.1007/s40368-025-01068-9

**Published:** 2025-10-03

**Authors:** S. Rajasekharan, N. Lygidakis, R. Cauwels, D. Declerck, J. Monteiro, S. Parekh

**Affiliations:** 1 Ghent University, Ghent, Belgium; 2 Private Dental Clinic, Athens, Greece; 3 KU Leuven, Leuven, Belgium; 4 Sheffield Teaching Hospitals NHS Foundation Trust, Sheffield, United Kingdom; 5 UCL Eastman Dental Institute, London, United Kingdom

**Keywords:** Antibiotics, Prophylaxis, Antimicrobial resistance

## Abstract

**Purpose:**

The European Academy of Paediatric Dentistry (EAPD) has developed this best clinical practice guidance on the use of antibiotics in pediatric dentistry, aiming to address challenges posed by antimicrobial resistance (AMR) and promote the responsible use of antibiotics.

**Methods:**

A comprehensive review of existing literature and global policies was conducted to evaluate antibiotic-prescribing practices for children. Recommendations were developed through a consensus process involving experts in pediatric dentistry during the EAPD policy workshop in Prague in May 2023.

**Results:**

The guidelines highlight the critical role of prudent antibiotic use to mitigate the growing threat of AMR. Specific recommendations include no routine antibiotic use for localized pulpitis or abscess without systemic involvement and targeted use for odontogenic infections with systemic signs. Detailed dosage protocols are provided, accounting for age, weight, and clinical context. Prophylaxis is recommended only for high-risk patients undergoing specific invasive procedures. Significant variations in national practices were noted, underlining the need for harmonized evidence-based protocols.

**Conclusion:**

These guidelines serve as a framework to optimize antibiotic use in children, emphasizing the importance of targeted prescribing practices and the implementation of preventive strategies in pediatric dental care.

## Introduction

Antibiotics were introduced in the 1940s, and have transformed modern medicine and saved millions of lives. Antibiotics have decreased the burden of infectious diseases markedly and have allowed the introduction of complex medical interventions such as major surgery, organ transplantations and care of premature babies, by preventing hospital-acquired infections (OECD 2015).However, overuse and misuse over time has resulted in global microbial resistance. The first cases of methicillin-resistant Staphylococcus aureus (MRSA) were already identified in the UK in 1962, and in the US in 1968 (Sengupta et al 2013; CDC 2013). Unfortunately, resistance has eventually been seen to nearly all antibiotics that have been developed (CDC 2013).

Antimicrobial resistance (AMR) is a global challenge and a priority for the European Union (EU). Each year, over 670,000 infections occur due to antibiotic-resistant bacteria in the European Union/European Economic Area (EU/EEA), and approximately 33,000 people die as a direct consequence of these infections. Efforts to prevent and control AMR are therefore vital for Europe (WHO 2017). Antimicrobial consumption is expressed as the average number of defined daily doses per 1000 inhabitants per day (DDD/1000). In 2021, the mean consumption of antimicrobials for systemic use in the community in 29 European countries was 15.0 (7.2–24.3) DDD per 1000 inhabitants per day. Higher levels of antimicrobial consumption were reported in the southern and eastern parts of the European Region, as compared to the north and west (ECDC 2022). Poor infection prevention and control practises (IPC) favour the further spread of these bacteria. Prudent antibiotic use and high standards of IPC in all health-care settings are therefore the cornerstones of an effective response to AMR.

## Survey of European guidelines

In order to get an insight/overview about current practises regarding the prescription of antibiotic therapy amongst paediatric dentists in Europe, a survey was undertaken in 2023. An e-mail was sent to each one of the 27 councillors of the European Academy of Paediatric Dentistry (EAPD), followed by two reminders, requesting information regarding currently used instructions on the prescription of antibiotics in the field of paediatric dentistry in their countries. This could be in the form of guidelines, best clinical practise or recommendations issued by National, European or International governmental authorities or by relevant professional organisations. The information gathered reported on indications and contraindications for antibiotic prescription, antibiotic of choice in each case, dosage, frequency, age range and route of administration.

Data were obtained from 23 out of 27 councillors (response rate: 85.2%). Specific guidelines were available in less than half of the countries (11 out of 23, 47.8% including Belgium, Denmark, Finland, France, Germany, Ireland, The Netherlands, Norway, Poland, Sweden and the UK). In three of the 11 countries (27.3%) there was a separate section for paediatric dentistry, whilst in the remaining countries this was integrated into recommendations for use in general dentistry. Twelve councillors reported that no country-specific guidelines were available (Croatia, Cyprus, Czech Republic, Greece, Israel, Italy, Romania, Russia, Slovenia, Serbia, Switzerland and Turkey).

Two specific clinical conditions were further explored: odontogenic abscess in a primary tooth and replantation of an avulsed permanent tooth. In case of the odontogenic abscess, respondents agreed unanimously that antibiotic therapy is only indicated when systemic signs of infection are present. In addition, when there is evidence of local spread of the infection, antibiotics are recommended if the child is immunocompromised or there is lack of cooperation for immediate intervention. Penicillin V was the antibiotic of first choice most frequently recommended (10 out of 18 respondents, 55.6%), with the dosage and duration showing great variation. Second choice products were mentioned in only a few guidelines (5 out of 18, 27.8%), with amoxicillin being advised in 60.0% of the cases (3 out of 5). In case of insufficient therapeutic response, combination therapy with metronidazole was also mentioned for 6 countries (33.3%). Dosage calculation varied in different guidelines, with some recommending a body weight-based method whilst others propose age-standardised doses. Regarding duration of antibiotic therapy, variation was large, ranging between 3 to 10 days, with 5 days being most frequently recommended (33.3%).

In case of replantation of an avulsed permanent tooth, information was available from 12 countries, with antibiotic therapy being recommended by 8 out of 12 (75.0%). This survey clearly highlights the need for updated guidelines, based on the most recent scientific evidence.

## Methods

An expert group (Dominique Declerck, Rita Cauwels, Sivaprakash Rajasekharan) was invited by EAPD to assess and summarise existing knowledge regarding antibiotic guidance. This best clinical practise recommendation has derived from both the review presented by the expert group during the 13th EAPD interim meeting seminar in Prague in May 2023 and the subsequent discussion in the working group, consisting of nominated delegates from EAPD member countries, moderated by members of the EAPD Clinical Affairs Committee (Nikos N. Lygidakis, Sivaprakash Rajasekharan). This workshop was held in May 2023 with a focus on:

- healthy patients;
- oral administration;
- as an adjuvant to (dental) treatment, not as a substitute to treatment.

It was agreed that clinical judgement and individual case interpretation remain important. Dose correction was needed in case of under/overweight children.

This guideline is an update of the previous EAPD guideline from 2002, with a focus on adjuncts to therapy of orofacial infection. The best clinical practise guidance was developed by the expert group and members of the Clinical Affairs Committee on behalf of the European Academy of Paediatric Dentistry.

## Recommendations

### Pulp infection

#### Localised pulp inflammation or infection without signs of systemic involvement

There is some evidence from adult studies that systemic antibiotic therapy for symptomatic apical periodontitis or acute apical abscess does not improve treatment outcomes (pain/swelling), in comparison to placebo (Cope et al. 2018). Antibiotics are not recommended for pulpitis or localised periapical periodontitis, in both primary and permanent teeth, as shown in Table 1.

**Table 1:** Recommendation for antibiotic prescription for symptomatic pulpitis in primary and permanent teeth and acute odontogenic abscess in combination with dental treatment and no systemic involvement

Recommendation	Strength of recommendation	Level of evidence
Symptomatic pulpitis in primary and permanent teeth No antibiotics recommended	Strong	Low/very low
Clinical practise guidance: treatment should commence as soon as possible		
Odontogenic abscess in combination with dental treatment and no systemic involvement No antibiotics recommended	Strong	Very low
Clinical practise guidance: treatment should commence as soon as possible		

This is a strong recommendation, even though the level of evidence is low. These recommendations are in agreement with other national and international guidelines for antibiotic therapy in paediatric dentistry and present no change to the previous EAPD guideline (Alaluusua et al. 2002).

#### Symptomatic pulp infection with signs of systemic involvement

Acute odontogenic infections with extra-oral swellings or cellulitis presenting as emergencies. Oral antibiotics are indicated when elevated temperature, evidence of systemic spread and local lymph node involvement are present (Palmer et al. 2020). In rapidly growing infections, hospital admission and intravenous administration of antibiotics may be necessary. Systemic signs and symptoms include pyrexia, malaise, difficulties breathing, trismus, dysphagia and/or dehydration (Palmer et al. 2020).

In all cases, adequate examination and imaging followed by immediate drainage or removal of the cause of infection (extraction) is advised, as shown in Table 2 (weak recommendation, very low evidence).

**Table 2:** Recommendation for antibiotic prescription in case of acute odontogenic abscess with systemic involvement

Recommendation Odontogenic abscess with systemic involvement	Strength of recommendation	Level of evidence
Antibiotics are recommended	Weak	Very low
Clinical practise guidance: treatment should commence as soon as possible		

##### Antibiotic regimens

Penicillins continue to be the antibiotic group of choice for the majority of odontogenic infections. Evidence showed that duration of treatment should be for up to 5 days, with recommended review after 2–3 days and discontinuation of therapy following the elimination of the source of infection and resolution of systemic signs/symptoms (Palmer et al. 2020). Antibiotic regimens should take in consideration the child’s age, weight and maximum daily dosage and allergy status. Country-specific antibiotic prescribing guidelines should be followed. In the absence of local guidelines, antibiotic regimen suggestions are presented in Appendix 1.

### Replantation of an avulsed permanent tooth

The administration of systemic antibiotics post-replantation of an avulsed tooth is controversial, with some authors advising its use to prevent or eliminate bacterial infection, help periodontal healing and pulpal revascularisation (Andersson et al. 2012), whilst others questioning their added value (Hinckfuss et al. 2009). The previous 2002 EAPD guidelines recommended the use of antibiotics following tooth replantation hoping to reduce the risk of external root resorption (Andreasen et al. 2000).

Α recent systematic review investigated three outcomes following replantation of an avulsed permanent tooth and the benefit of antibiotics: tooth survival (critical outcome), and periodontal ligament healing and pulpal healing (important outcomes). Evidence of very low quality suggests that the use of systemic antibiotics at replantation of avulsed permanent teeth is not associated with improved tooth survival, periodontal ligament healing or pulpal healing (Leroy et al 2020). For this reason, the authors concluded that antibiotics should not be prescribed following replantation of an avulsed tooth due to the lack of evidence suggesting otherwise, as shown in Table 3.

**Table 3:** Recommendation for antibiotic prescription following replantation of an avulsed permanent tooth

Recommendation	Strength of recommendation	Level of evidence
The administration of systemic antibiotics is not recommended	Strong	Very Low

### Periodontitis and necrotising periodontal diseases

It has been suggested that systemic antimicrobials may have a role in the (non-surgical or Step 2) treatment of periodontitis, but their use should be restricted to certain patients and certain periodontal conditions (Herrera et al. 2002). A recent systematic review, with data from former aggressive periodontitis cases (Leroy et al 2020), included in the original systematic review by Teughels et al 2020, investigated the benefit of antibiotics as an adjunct to subgingival instrumentation, in step 2 of the treatment of periodontitis in the permanent dentition by analysing the following critical outcomes: tooth survival, pocket closure; and the following important outcomes: full mouth clinical attachment level, full mouth probing depth, bleeding on probing, residual pockets with probing depth of > 5 mm (Teughels et al 2020). The authors found low quality evidence showing that the use of antibiotics did not improve the critical outcomes. However, evidence of low/moderate quality showed an improvement in all important outcomes after antibiotic administration, as shown in Table 5 (Leroy et al 2020).

Necrotising periodontal diseases, in temporarily and/or moderately immune-compromised patients, are characterised by gingival necrosis, bleeding and pain (Herrera et al 2018). In severe cases, systemic signs and symptoms such as lymphadenopathy, fever, and malaise may be present. Local therapeutic measures with adequate pain control provide resolution of the acute phase of necrotizing gingivitis (Palmer et al 2020) (Table 4). Antibiotic regimen suggestions are presented in Appendix 1.

**Table 4:** Recommendation for antibiotic prescription in case of non-surgical treatment of periodontitis

Recommendation	Strength of recommendation	Level of evidence
Periodontitis -The use of systemic antibiotics in combination with the non-surgical/ step 2 treatment of aggressive/ grade C periodontitis can be considered	Weak	Low
Necrotising periodontal diseases—Antimicrobials are recommended only as an adjunct to local measures where there is evidence of systemic involvement	Strong	Very low

For necrotising periodontal diseases in chronically, severe immune-compromised patients, a different approach may be followed, as the cases are also affected by severe nutritional deficiencies, extreme living conditions and co-morbidities (Herrera et al 2018).

### Pericoronitis

In line with the management of acute dental infections, local inflammation and infection is managed with local measures (extraction or operculectomy and/or incision and drainage where necessary). When there is evidence of systemic involvement, antimicrobials should be provided as an adjunct to local measures (Leroy et al 2020) (Table 5). Antibiotic regimen suggestions are presented in Appendix 1.

**Table 5:** Recommendation for antibiotic prescription in case of pericoronitis

Recommendation Pericoronitis	Strength of recommendation	Level of evidence
The use of antibiotics is not recommended in patients without systemic involvement and/or following adequate (periodontal) treatment	Strong	Very low
The use of antibiotics can be considered in patients with systemic involvement	Weak	Very low

### Antibiotic prophylaxis

The guidelines on antibiotic prophylaxis are purely for the purpose of guidance, and practitioners are encouraged to exercise their own clinical judgement and, when in doubt, assess patients on an individual basis. Individual patient circumstances may vary, and the information contained in this document may not cover all possible scenarios. It is therefore recommended that dentists consult a cardiologist in a timely manner to address specific concerns or uncertainties related to patient care. Maintenance of optimal oral hygiene should be strongly motivated as a general preventive measure for patients with high risk of infective endocarditis (IE). Patients should be encouraged to brush their teeth and use interdental cleaning aids (twice a day) and to seek professional dental cleaning and follow-up (at least twice yearly). In the absence of local guidelines, an antibiotic prophylaxis regimen suggestion is presented in Appendix 2.

In accordance with recent European Society of Cardiology (ESC) guidelines (Delgado et al 2023), the EAPD consensus agreement proposes that antibiotic prophylaxis is recommended in patients at high risk of IE undergoing at-risk dental procedures and is not currently recommended in other situations. For a clear understanding of this statement, it is necessary to list the patients who are at high risk of IE and which treatment procedures by dentists are considered as risky dental procedures (Tables 6 and 7).

**Table 6:** Antibiotic prophylaxis based on patient risk

Antibiotic prophylaxis is recommended	Antibiotic prophylaxis should be considered	Antibiotic prophylaxis may be considered
Patients with previous history of infective endocarditis	Patients with transcatheter mitral and tricuspid valve repair	Recipients of heart transplant
Patients with surgically implanted prosthetic valves with any material used for surgical cardiac valve repair		
Patients with transcatheter implanted aortic and pulmonary valvular prostheses		
Patients with congenital heart disease (CHD) (not including isolated congenital valve abnormalities)		
Patients with ventricular assist devices

**Table 7:** Antibiotic prophylaxis based on the dental treatment

Antibiotic prophylaxis is recommended for high-risk dental procedures
Dental extractions
Oral surgery procedures (including periodontal surgery and oral biopsies)
Perforation of the oral mucosa (excluding local anaesthesia)
Dental procedures involving manipulation of the gingival or periapical region of the teeth (including scaling and root canal treatment)

## Data Availability

No datasets were generated or analysed during the current study.

## Appendix 1

Appendix 1 Recommended antibiotic regimen as per EAPD consensus agreement

As antibiotics regimens vary according to country and different local guidelines, the recommended EAPD regimen is added as an appendix for information.

**Table:** Pulp infection and pericoronitis

Situation	Antibiotic	Regimen	Frequency
Standard – broad spectrum antibiotic	Amoxicillin or Penicillin V	75–100 mg/kg body weight/day 50–75 mg/kg body weight/day	in 3 doses, for 5 days
Penicillin allergy	Azithromycin or alternative	10 mg/kg body weight/day	in 1 dose, for 3 days
In severe infections, as an adjunct or substitute	Metronidazole	22.5 mg/kg body weight/day	in 3 doses, for 5 days

*Maximum adult dose should not be exceeded

**Table:** Periodontitis

Situation	Antibiotic	Regimen	Frequency
Standard	Amoxicillin and Metronidazole	*50 mg/kg body weight/day 30 mg/kg body weight/day*	in 3 doses, for 5—7 days
Allergy to penicillin	Metronidazole	*30 mg/kg body weight/day*	in 3 doses, for 5—7 days

*Maximum adult dose should not be exceeded

**Table:** Necrotizing periodontal diseases

Situation	Antibiotic	Regimen	Frequency
Standard	Metronidazole	*30 mg/kg body weight/day*	in 3 doses, for up to 5 days
If Metronidazole contraindicated	Amoxicillin	*50 mg/kg body weight/day*, increased in severe infections	in 3 doses, for 5—7 days

*Maximum adult dose should not be exceeded

## Appendix 2

Appendix 2 Recommended antibiotic prophylactic regimen for the prevention of endocarditis, as per EAPD consensus agreement

A single dose of antibiotics, 30–60 min before the dental procedure is advised.

**Table:**

	Antibiotic	Dosage	Route of administration
Standard	Amoxicillin	50 mg/kg	Orally
	Ampicillin	50 mg/kg	Intravenous or Intramuscular
Penicillin allergy	Cefazolin or ceftriaxone	50 mg/kg	Intravenous or Intramuscular
	Cephalexin	50 mg/kg	Orally
	Azithromycin or Clarithromycin	15 mg/kg	Orally
	Doxycycline	2.2 mg/kg (< 45 kg) 100 mg (> 45 kg)	Orally

*Maximum adult dose should not be exceeded

Oral streptococci are the main target for antibiotic prophylaxis. The risk of adverse fatal/non-fatal events appear to be high for clindamycin (mainly related to *Clostridioides difficile* infections). Hence, in agreement with the recommendations of the European Society of Cardiology, the use of clindamycin is not recommend for antibiotic prophylaxis. This is a notable change compared to the previous EAPD guideline published in 2002.
